# Supplementary material for: Spin diffusion from an inhomogeneous quench in an integrable system
Source: Nat Commun. 2017 Jul 13;8:16117. doi: 10.1038/ncomms16117 (PMC5554798; doi:10.1038/ncomms16117)
Supplement: Supplementary Information [file ncomms16117-s1.pdf]

File Name: Supplementary Information
Description: Supplementary Figures, Supplementary Notes and Supplementary References

File Name: Peer Review File
Description:

Supplementary Note 1 – Diffusive regime

We direct the readers attention to Supplementary Figure 1, where the local-time exponent α is shown as a function of time for several values of the anisotropy in the massive regime, namely $\Delta = 1.05, 1.1, 1.3, 1.5$. The asymptotic scaling exponent converges on the accessible time scale to $\alpha = 1/2$ – expect for the case $\Delta = 1.05$ where the convergence has not yet been reached though the extrapolated asymptotic value is likely the same – is distinctly different from the exponent $\alpha = 2/3$ for the isotropic case $\Delta = 1$.

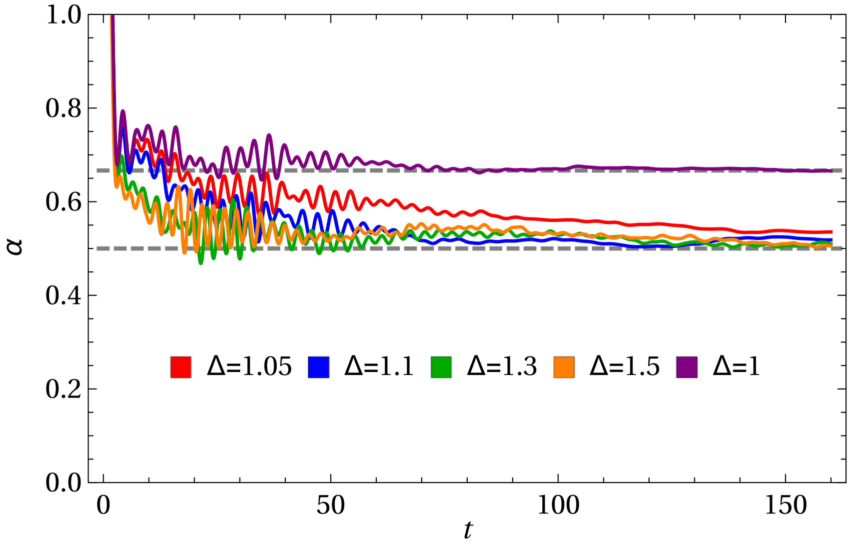

Supplementary Figure 1: **Scaling exponents.** We show the time dependence of the scaling exponent α for various values of the anisotropy Δ in the massive regime, as well as for the isotropic reigme $\Delta = 1$ for comparison. Nearer to $\Delta = 1$ the time scale at which the final value is reached appears to increase but the convergence trend is clear.

Supplementary Note 2 – Ballistic regime

Spin transport is known to be ballistic in the massless regime, $\Delta < 1$. Here we verify that the chosen inhomogeneous quench does indeed reproduce this result. In Supplementary Figure 2 we demonstrate the convergence of local-time scaling exponent α for a few cases $\Delta = 0, 0.5, 0.7, 0.9$ to an asymptotic ballistic exponent $\alpha = 1$, and again compare it to the isotropic case $\Delta = 1$.

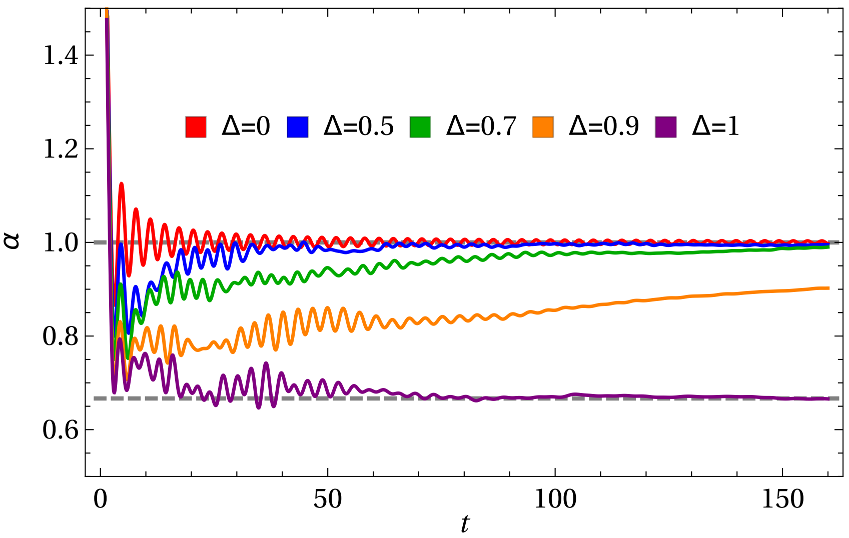

Supplementary Figure 2: **Scaling exponents.** We show the time dependence of the scaling exponent α for various values of the anisotropy Δ in the massless regime. We observe clear asymptotic convergence towards the ballistic value α, while the convergence times increase when Δ approaches 1, however the transition to $\Delta = 1$ – shown for comparision – appears to be discontinuous.

In Supplementary Figure 3 we also show the scaling of spin and current density profiles which clearly exhibit expected ballistic behaviour [1, 2]. In the non-interacting case $\Delta = 0$ we find also excellent agreement with analytic solutions [3].

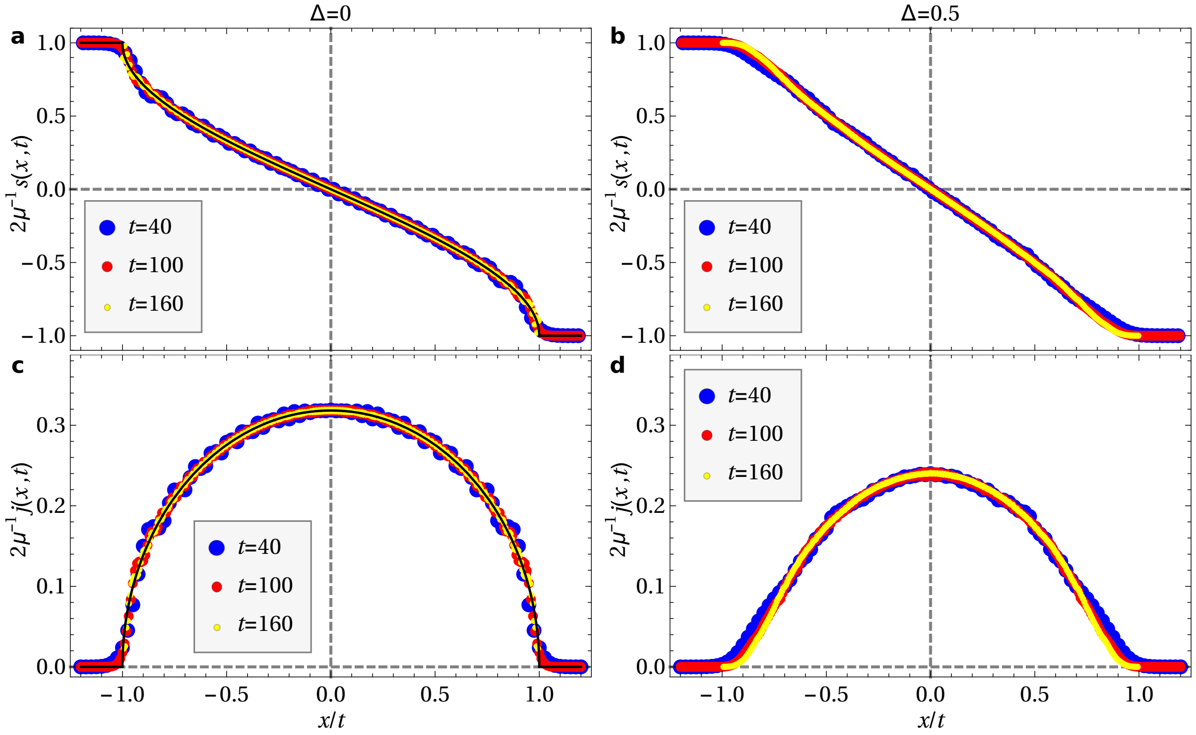

Supplementary Figure 3: **Scaling functions.** We show spin (a and b) and current (c and d) density profiles for two values of the anisotropy $\Delta = 0$ (left) and $\Delta = 0.5$ (right) with respect to a single scaling variable x/t suggestive of ballistic transport expected for this regime. In the non-interacting case $\Delta = 0$ we also indicate known analytic solutions which excellently match the numerical data.

Supplementary References

[1] O. A. Castro-Alvaredo, B. Doyon and T. Yoshimura, Emergent hydrodynamics in integrable quantum systems out of equilibrium, *Phys. Rev. X* **6**, 041065 (2016).

[2] B. Bertini, M. Collura, J. De Nardis, M. Fagotti, Transport in out-of-equilibrium XXZ chains: exact profiles of charges and currents, *Phys. Rev. Lett.* **117**, 207201 (2016).

[3] T. Antal, Z. Racz, A. Rakos, G. M. Schütz, Transport in the XX chain at zero temperature: Emergence of flat magnetization profiles, *Phys. Rev. E* **59**, 4912-4918 (1999).
